# Biochemical Biomarkers and Neurodegenerative Diseases

**DOI:** 10.3390/brainsci11070940

**Published:** 2021-07-16

**Authors:** Marcello Ciaccio

**Affiliations:** 1 Institute of Clinical Biochemistry, Clinical Molecular Medicine and Laboratory Medicine, Department of Biomedicine, Neurosciences, and Advanced Diagnostics, University of Palermo, 90127 Palermo, Italy; marcello.ciaccio@unipa.it; 2 Department of Laboratory Medicine, AOUP “P. Giaccone”, 90127 Palermo, Italy

Neurodegenerative diseases (ND) are a heterogeneous group of disorders characterized by progressive dysfunction and loss of neurons in different areas of the central nervous system or peripheral nervous system. NDs, including Alzheimer’s disease (AD), Parkinson’s disease (PD), and motor neuron disease (MND), represent a big challenge for scientific research due to their prevalence, cost, basic pathophysiological mechanisms, and lack of mechanism-based treatments. The diagnosis, prognosis, and monitoring of such disorders are complex and rely mainly on clinical criteria. In the last decades, biochemical markers have emerged as promising tools in the field of ND. The articles belonging to this Special Issue of “Biochemical Biomarkers and Neurodegenerative Disorders” encompass the last literature evidence on the importance of biomarkers in the management of ND, from screening to diagnosis, prognosis, and treatment.

Scazzone et al. explored the association among Vitamin D3, single nucleotide polymorphisms (SNPs), and Multiple Sclerosis (MS) in a retrospective case-control study [1]. They showed that MS patients had significantly lower levels of Vitamin D3 than controls, but no association among SNPs, Vitamin D3, and MS risk was found. The role of hypovitaminosis D in MS risk has been widely investigated in the last decades, and some literature evidence supports the hypothesis that Vitamin D3 could be involved in MS pathogenesis. Noteworthy, Vitamin D3 status is influenced by both genetic and environmental factors. Thus, many Authors investigated the possible influence of genetic variants in Vitamin D3 related genes on MS risk, achieving contrasting results [2].

Beyond its well-known role in calcium homeostasis, Vitamin D3 has pleiotropic functions, including immune-regulation and neurological function [3]. Thus, its possible role as a biomarker or risk factor in several autoimmune and neurodegenerative diseases has been evaluated. Bivona et al. described the current knowledge on the role of Vitamin D3 in Alzheimer’s Disease (AD), stating that a definite conclusion cannot be drawn because controversial findings have been found across the studies [4].

Another interesting area of research is the role of circulating biomarkers in Inherited Neuromuscular Disorders (INMD), defined as a heterogeneous group of genetic diseases characterized by progressive muscle degeneration and weakness and associated with long-term disability. They represent rare disorders whose diagnosis is based on an extensive clinical evaluation with complementary genetic analysis. Due to the presence of genetic heterogeneity and lack of segregation in sporadic cases, a definite diagnosis is challenging. Thus, serum biomarkers are strongly sought after. Lupica et al. described several promising biomarkers that could help clinicians in the diagnostic workup of INMD [5].

Another rare disease with important clinical consequences is Amyotrophic Lateral Sclerosis (ALS). Many efforts are ongoing to find prognostic biomarkers of this devastating disease. Colletti et al. found that beta-amyloid 1–42 (Aβ 1–42) could be involved in the pathogenesis of ALS, and the Aβ 1–42/Aβ 1–40 ratio could represent a biomarker of prognosis [6].

Finally, an interesting article was focused on the current COVID-19 pandemic, raising the question if SARS-CoV-2 infection could induce long-term neurological consequences [7]. Notable, SARS-CoV-2 is a neurotropic virus and, consequently, it could predispose and accelerate the development of neurological disorders, such as AD. However, we may have the answer to such an interesting question in the next few years.
